# Fibroblast growth factor 23 and calcium‐phosphate metabolism in relation to cardiovascular risk factors in patients with type 1 diabetes

**DOI:** 10.1111/1753-0407.13500

**Published:** 2023-12-20

**Authors:** Stephanie Vermeulen, Mirjam E. A. Scheffer‐Rath, Martine T. P. Besouw, Amarens van der Vaart, Martin H. de Borst, Annemieke M. Boot

**Affiliations:** ^1^ Department of Pediatric Endocrinology, Beatrix Children's Hospital University Medical Center Groningen, University of Groningen Groningen the Netherlands; ^2^ Diabeter Center for Pediatric and Adolescent Diabetes Care and Research Groningen the Netherlands; ^3^ Department of Pediatrics Rijnstate Hospital Arnhem the Netherlands; ^4^ Department of Pediatric Nephrology, Beatrix Children's Hospital University Medical Center Groningen, University of Groningen Groningen the Netherlands; ^5^ Department of Internal Medicine, Division of Nephrology University Medical Center Groningen, University of Groningen Groningen the Netherlands; ^6^ Department of Internal Medicine, Division of Endocrinology University Medical Center Groningen, University of Groningen Groningen the Netherlands

**Keywords:** calcium, diabetes mellitus type 1, FGF23, phosphate, smoking

## Abstract

**Background:**

Cardiovascular disease (CVD) is the major cause of mortality in type 1 diabetes (T1D). The objective of this study is to evaluate fibroblast growth factor 23 (FGF23) and calcium‐phosphate metabolism in relation to cardiovascular risk factors in adults with and without T1D.

**Methods:**

A case–control study was conducted using data from patients with T1D and age‐ and sex matched controls without T1D from the Lifelines Cohort Study.

**Results:**

We included 302 adults in the T1D group and 302 adults in the control group. Median age was 42 years. Median glycosylated hemoglobin (HbA1c) in the T1D group was 7.8%. FGF23 of all patients with T1D was not significantly different from controls. Females with T1D had significantly higher FGF23 than males with T1D (83.3 vs 69.3 U/mL, *p* = 0.002), this was not observed in controls. Serum phosphate, calcium, and alkaline phosphatase were higher and parathyroid hormone was lower in patients with T1D, compared to controls (all *p* < .001), all within normal range. In the T1D group, FGF23 was positively correlated with serum phosphate (*p* < .001), alkaline phosphatase (*p* = .01), and calcium (*p* = .030), these correlations were not observed in controls. Median FGF23 was significantly higher in current smokers than in nonsmokers with T1D (84.9 vs 73.5 U/mL, *p* < .05).

**Conclusions:**

Serum calcium, phosphate, and alkaline phosphatase were higher in patients with T1D than in controls and were positively correlated to FGF23 in patients with T1D. Current smokers with T1D had higher FGF23 than nonsmokers with T1D. These findings may contribute to the increased risk of CVD in patients with T1D.

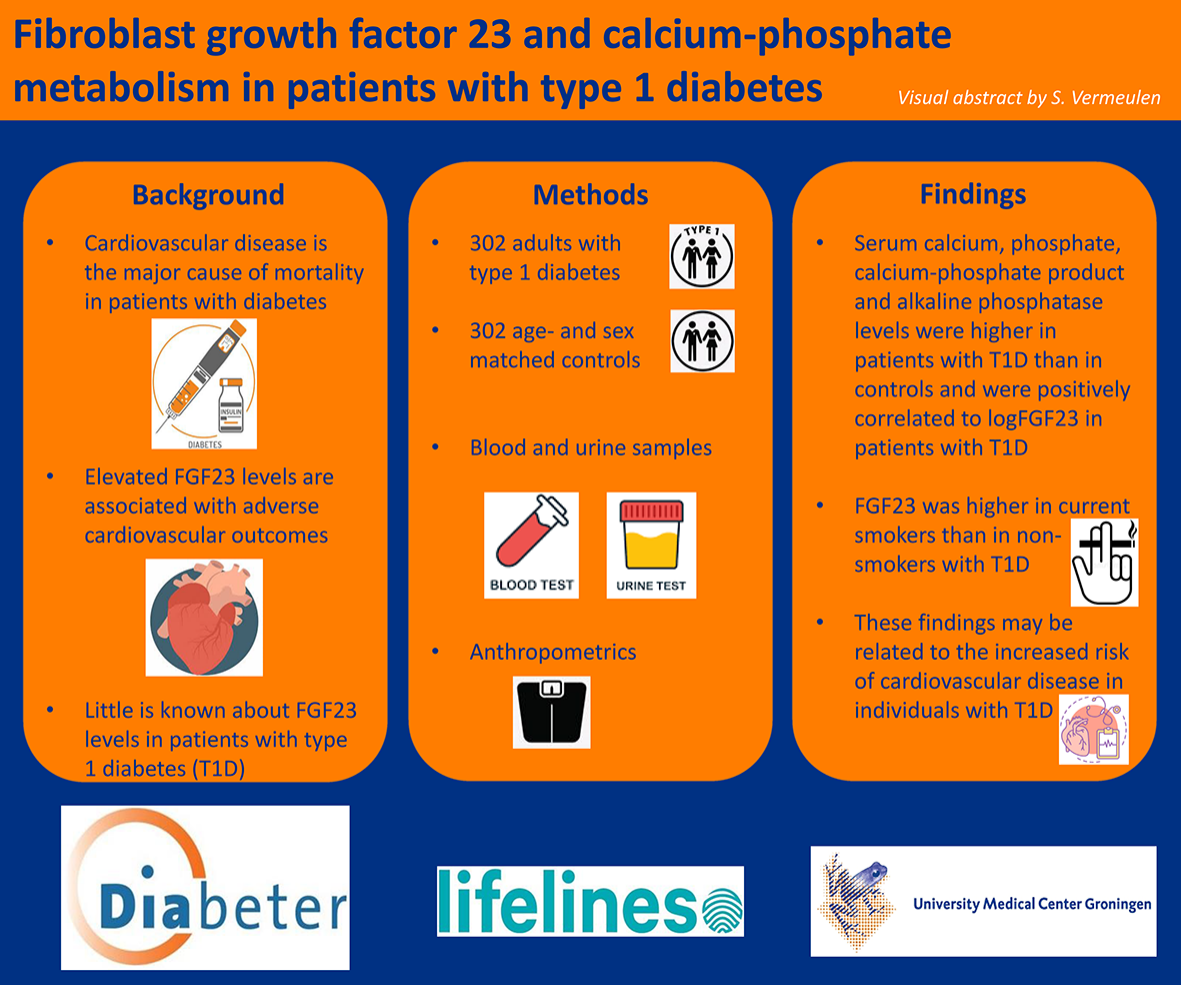

## Graphical abstract

Highlights

- Serum calcium, phosphate, calcium‐phosphate product and alkaline phosphatase levels were significantly higher in adult patients with type 1 diabetes (T1D) than in controls and were positively correlated to fibroblast growth factor 23 (FGF23) in patients with T1D. FGF23 was higher in current smokers than in nonsmokers with T1D. These findings may be related to the increased risk of cardiovascular disease in individuals with T1D.

## 1 INTRODUCTION

Diabetes mellitus is an important global health problem. In 2021 the International Diabetes Federation estimated that around 537 million adults were living with diabetes.^1^ It was estimated that, in 2021, there were about 8.4 million individuals worldwide with type 1 diabetes (T1D).^2^ Cardiovascular disease (CVD) is the major cause of mortality in the population with diabetes.^3^ In patients with T1D CVD typically becomes clinically apparent in adulthood, although the pathogenesis begins at disease onset.^4^, ^5^ Morbidity and mortality due to CVD increase with a younger age of onset and a longer disease duration. Many factors contributing to CVD have been identified. These include classical cardiovascular risk factors such as smoking, obesity, hypertension, and dyslipidemia, whereas hyperglycemia in itself is also an important risk factor of CVD. Even though current treatment regimens target these risk factors, the overall burden of CVD in diabetes remains high. For example, the life expectancy for the “average” person with T1D is still 7.6 years lower compared to the “average” person in the nondiabetic population.^6^ This stimulates research to other contributing risk factors and possible new treatment options.

Preventing hyperphosphatemia is important since it is associated with CVD and mortality.^7^ Fibroblast growth factor 23 (FGF23) is the main phosphate‐regulating hormone.^8^, ^9^, ^10^ FGF23 is primarily synthesized in bone and its main target organs are the kidneys. FGF23 stimulates renal phosphate excretion by inhibiting phosphate reabsorption in the proximal tubules and decreases gastrointestinal phosphate absorption by inhibiting the synthesis of active vitamin D. Secretion of FGF23 is regulated by local and systemic factors, including calcium, phosphate, vitamin D, parathyroid hormone (PTH), acidosis, and inflammatory cytokines.^11^

FGF23 has a role in cardiovascular risk in CKD^12^, ^13^, ^14^ and possibly in glucose metabolism.^15^, ^16^, ^17^, ^18^, ^19^, ^20^ FGF23 may have an important role in development of atherosclerosis and vascular dysfunction in general population^21^, ^22^ and in the development of left ventricular hypertrophy and reduced ejection fraction in patients with CKD.^10^, ^12^ In patients with CKD, higher levels of FGF23 are associated with progression of kidney disease and mortality independent of other risk factors.^13^, ^14^ Previous studies have found that elevated FGF23 levels are associated with adverse cardiovascular outcomes in patients with and without established CVD.^15^, ^16^, ^17^ Several studies have demonstrated that FGF23 has direct effects on cardiomyocytes. FGF23 activates fibroblast growth factor receptor 4 (FGFR4) phospholipase Cγ/calcineurin/nuclear factor of activated T cells signaling in cardiomyocytes and leads to cardiac hypertrophy and myocardial fibrosis in rodents.^23^ FGF23 also has an indirect effect on the cardiomyocytes leading to hypertrophy and fibrosis. High levels of FGF23 lead to lower levels of active vitamin D, which activate the renin‐angiotensin‐aldosterone system causing cardiac hypertrophy and fibrosis. Circulating FGF23 also causes an increase in local angiotensinogen and angiotensin II expression in cardiomyocytes, leading to hypertrophy and fibrosis.^23^, ^24^

Interestingly, FGF23 may also play a role in glucose metabolism. Previous studies showed a positive correlation between insulin resistance and FGF23 levels in patients with CKD.^18^, ^19^ Little is known about FGF23 levels in patients with T1D. In a previous study in 68 patients with T1D without previous cardiovascular events, higher FGF23 levels were positively associated with arterial stiffness measured as aortic pulse wave velocity, also after correction for several bone mineral parameters, estimated glomerular filtration rate (eGFR) and classical cardiovascular risk factors.^25^ Only few general population studies were performed and the following factors were associated with higher FGF23 levels: female sex, smoking, parathyroid hormone levels, GFR, C‐reactive protein, higher serum calcium levels, higher energy intake, lower iron intake, adiposity, and dyslipidemia.^14^, ^26^, ^27^, ^28^

It is not entirely clear whether FGF23 is merely an indicator of disease or actively contributes to disease progression and therefore could be a therapeutic target. The objective of this study is to evaluate FGF23 levels and calcium‐phosphate metabolism in relation to cardiovascular risk factors in adults with and without T1D.

## 2 METHODS

### 2.1 Study design

We used data from the Lifelines Cohort Study. Lifelines is a multidisciplinary prospective population‐based cohort study examining in a unique three‐generation design the health and health‐related behaviors of 167 729 persons living in the north of the Netherlands. It employs a broad range of investigative procedures in assessing the biomedical, sociodemographic, behavioral, physical, and psychological factors that contribute to the health and disease of the general population, with a special focus on multimorbidity and complex genetics. Lifelines works with a combination of questionnaires, comprehensive physical examinations, and biological samples; providing a unique source for research purposes. Biological samples are stored at −80°C to ensure high quality and long‐term preservation. Detailed information regarding recruitment strategy and the representativeness of the Lifelines study population has been documented elsewhere.^29^, ^30^

The Lifelines Cohort Study is conducted according to the principles of the Declaration of Helsinki and the research code of the University Medical Center Groningen (UMCG). The Lifelines protocol was approved by the UMCG Medical ethical committee under number 2007/152. All participants gave written informed consent to participate in the study.

### 2.2 Study population

In this study, all patients with T1D (based on self‐reporting in questionnaires) who were included at baseline in the Lifelines database (between 2006 and 2013) were eligible for inclusion (*n* = 304). We excluded two patients from the T1D group because they either reported no use of insulin or had an age of onset of diabetes >50 years. The age‐ and sex‐matched controls were derived from the same database, leaving us with a final study population of 604 subjects.

### 2.3 Clinical measurements

Blood samples were collected at baseline by venipuncture. Serum levels of glucose, HbA1c, total cholesterol (TC), high‐density lipoprotein cholesterol (HDL‐c), low‐density lipoprotein cholesterol (LDL‐c), triglycerides (TG), calcium, phosphate, alkaline phosphatase, creatinine, and eGFR (CKD Epidemiology Collaboration formula 2009) were analyzed directly. From serum calcium and serum phosphate levels the calcium‐phosphate product (CPP) was calculated (calcium × phosphate = CPP). Plasma levels of FGF23, intact PTH, and 25‐hydroxy vitamin D (25(OH)D) were analyzed later. Plasma c‐terminal FGF23 was determined by ELISA of TECO Medical. PTH was assessed by immunoassay of Roche Cobas. 25(OH)D was assessed by liquid chromatography with tandem mass spectrometry. Urine samples were collected in the 24 h before collection of the blood samples. Levels of albumin and creatinine were measured in urine, after which albumin/creatinine ratios (UACR) were calculated.

Baseline measurements of blood pressure and anthropometry were performed by trained research staff following standardized protocols. Anthropometric measurements were performed without shoes and heavy clothing. According to their body mass index (BMI) patients were classified as having a normal BMI (BMI range 18–25 kg/m^2^), being overweight (BMI range 25–30 kg/m^2^), or being obese (BMI >30 kg/m^2^). Participants were considered to have hypertension at baseline if they used antihypertensive medication, had systolic blood pressure ≥140 mm Hg and/or had diastolic blood pressure ≥ 90 mm Hg. Dyslipidemia was defined as the use of a statin, a TC level >5.0 mmol/L, an HDL‐c level <1.0 mmol/L, an LDL‐c level >2.6 mmol/L for the T1D group and >3.0 mmol/L for the control group or a TG level >2.0 mmol/L. Impaired kidney function was defined as an eGFR <60 mL/min/1.73 m^2^. In line with the American Diabetes Association guidelines elevated UACR was defined as an UACR >3.0 mg/mmol (>30 mg/mg).

### 2.4 Statistical analysis

Statistical software (Statistical Package for the Social Sciences [SPSS] for Windows, version 25, SPSS, Inc., Chicago, IL) was used for statistical analysis. The normal distribution of variables was assessed with normal Q‐Q plots and detrended Q‐Q plots. Normally distributed values are presented as mean ± SD and skewed variables as median [interquartile range]. Categorical variables are presented as absolute numbers (%). Differences in means between groups were tested using the Student's *t* test. Differences in medians between groups were tested using the Mann–Whitney *U* test. Correlations were analyzed using Spearman's rank. For univariate and multiple regression analyses log transformation was applied for FGF23, TC, and TG to achieve normal distribution. Covariates were selected for multivariable models if significance was observed in the Spearman correlation test or univariate correlation analysis. For multiple regression analyses the enter method was used. Statistical significance was defined as *p* < .05.

## 3 RESULTS

Our study population (*n* = 604) was divided into two groups. Both the T1D group as the control group consisted of 302 adults (50% male). The median age in both groups was 42 years (range 18–76 in T1D group and 18–73 in control group). Median HbA1c for the T1D group was 7.8% (62 mmol/mol). For 301 subjects in the T1D group time since T1D diagnosis was documented. Median time since T1D diagnosis was 20 years. Table 1 presents the demographic characteristics of the study population.

**TABLE 1:** Demographic characteristics.

	T1D group (*n* = 302)	Control group (*n* = 302)	*p* value
General data			
Sex (male/female)	151/151	151/151	
Age (year)	42 (35–48)	42 (35–48)	.942
BMI (kg/m^2^)	25.6 (23.2–28.3)	25.0 (22.9–27.5)	.053
Heart rate (/min)	75 (66–83)	72 (64–80)	**<.001**
Systolic blood pressure (mm Hg)	125 (117–135)	122 (114–132)	**.003**
Diastolic blood pressure (mm Hg)	71 (67–77)	71 (67–78)	.915
Smokers	65 (22%)	72 (24%)	
Pack years of smokers	6.4 (0.7–17)	5.8 (0.6–12.9)	.643
Use of ACE inhibitors	79 (26%)	<10 (<3%)	
Use of statins	93 (31%)	<10 (<3%)	
Glucose metabolism			
Duration of diabetes (year)	20 (10–28)	N/A	
HbA1c (%)	7.8 (7.1–8.5)	5.6 (5.4–5.8)	**<.001**
Biochemical data			
FGF23 (U/mL)	76.4 (57.7–97.7)	72.9 (58.5–93.4)	.522
Phosphate (pmol/L)	1.04 (SD 0.25)	0.89 (SD 0.16)	**<.001**
Calcium (mmol/L)	2.32 (2.26–2.39)	2.27 (2.22–2.32)	**<.001**
CPP (mmol^2^/L^2^)	2.39 (SD 0.61)	2.01 (SD 0.37)	**<.001**
Alkaline phosphatase (mmol/L)	72 (59–88)	59 (49–73)	**<.001**
25(OH)D (nmol/L)	52 (37–68)	56 (43–67)	.176
PTH (mmol/L)	3.4 (2.8–4.4)	3.9 (3.1–5.0)	**<.001**
eGFR (mL/min/1.73 m^2^)	99 (82–118)	102 (86–120)	.099
UACR (mg/mmolCr)	0.31 (0.15–0.66)	0.27 (0.16–0.50)	.427
Lipid profile			
LDL‐c (mmol/L)	2.6 (2.2–3.0)	3.1 (2.5–3.7)	**<.001**
HDL‐c (mmol/L)	1.5 (1.2–1.9)	1.4 (1.2–1.7)	**<.001**
TG (mmol/L)	0.8 (0.6–1.1)	0.9 (0.7–1.3)	**<.001**
TC (mmol/L)	4.4 (4.0–4.9)	4.9 (4.4–5.5)	**<.001**

*Note*: Data are in *n* (%), mean (SD) or median (25th quartile–75th quartile). Bolded values are *p* < .05.

Abbreviations: 25(OH)D, 25‐hydroxy vitamin D; ACE, angiotensin‐converting enzyme; BMI, body mass index; CPP, calcium‐phosphate product; eGFR, estimated glomerular filtration rate; FGF23, fibroblast growth factor 23; HbA1c, glycosylated hemoglobin; HDL‐c, HDL‐cholesterol; LDL‐c, LDL‐cholesterol; PTH, parathyroid hormone; T1D, type 1 diabetes; TC, total cholesterol; TG, triglyceride; UACR, urine, after which albumin/creatinine ratios.

### 3.1 FGF23 and calcium‐phosphate metabolism

Median FGF23 in patients with T1D was not significantly different from controls. When we focused on the T1D group we did find that females with T1D had significantly higher FGF23 than males with T1D (83.3 vs 69.3 U/mL, *p* = .002), this was not observed in controls (75.6 vs 69.2 U/mL, *p* = .358).

Serum phosphate, calcium, CPP, and alkaline phosphatase levels were higher and PTH levels were lower in patients with T1D compared to controls (all *p* < .001), although all were within normal range. 25(OH)D in patients with T1D was not significantly different from controls (Table 1).

Univariate regression analysis showed that sex (*r* = 0.177, *p* = .002), HbA1c levels (*r* = 0.040, *p* < .001), serum phosphate (*r* = 0.236, *p* < .001), calcium (*r* = 0.125, *p* = .030), CPP (*r* = 0.236, *p* < .001), alkaline phosphatase (*r* = 0.201, *p* < .001), and PTH (*r* = 0.122, *p* = .035) were significantly associated with logFGF23 levels in the T1D group. We did not find these results in the control group (Table 2).

**TABLE 2:** Univariate correlation analysis of logFGF23 and other parameters.

	T1D group		Control group	
	*r*	*p* value	*r*	*p* value
Age	0.036	.538	0.046	.421
Sex	0.177	**.002**	0.039	.495
Smoking	0.076	.191	0.011	.852
BMI	0.016	.782	0.090	.118
Time since T1D diagnosis	0.011	.853	n/a	n/a
HbA1c	0.040	**<.001**	0.021	.715
Phosphate	0.236	**<.001**	0.023	.688
Calcium	0.125	**.030**	0.029	.617
CPP	0.236	**<.001**	0.026	.649
Alkaline phosphatase	0.201	**<.001**	0.064	.266
PTH	0.122	**.035**	0.007	.910
25(OH)D	0.017	.768	0.055	.342
LogTC	0.081	.159	0.001	.982
LogTG	0.100	.082	0.019	.736

*Note*: Bolded values are *p* < .05.

Abbreviations: 25(OH)D, 25‐hydroxy vitamin D; BMI, body mass index; CPP, calcium‐phosphate product; FGF23, fibroblast growth factor 23; HbA1c, glycosylated hemoglobin; PTH, parathyroid hormone; T1D, type 1 diabetes; TC, total cholesterol; TG, triglyceride.

### 3.2 FGF23 and cardiovascular disease risk

#### 3.2.1 Smoking

In the T1D group 65 of the 302 subjects reported to be current smokers versus 72 of the 302 subjects in the control group (22% vs 24%, respectively). Median FGF23 in the T1D group was significantly higher in current smokers than in nonsmokers (84.9 vs 73.5 U/mL, *p* < .05). This difference was not present in the control group. The group of current smokers with T1D consisted of 36 males and 29 females. The nonsmokers with T1D consisted of 115 males and 120 females. The analyses for males and females separately showed no statistical significance. FGF23 was not significantly correlated with pack years in both groups. In the univariate analysis logFGF23 was not significantly correlated with smoking in the T1D group nor in the control group.

Serum calcium, phosphate, and CPP in current smokers were not significantly different from nonsmokers in patients with T1D nor in the control group. Serum phosphate, calcium, and CPP were not significantly associated with pack years.

#### 3.2.2 Obesity

In the T1D group 132 subjects (44%) had a normal BMI, 123 subjects (41%) were overweight, and 47 subjects (15%) were obese. In the control group 150 subjects (50%) had a normal BMI, 121 subjects (40%) were overweight, and 31 subjects (10%) were obese. In both groups there were no subjects with a BMI < 18 kg/m^2^. Median BMI in the T1D group was not significantly different from the control group (25.6 vs 25.0 kg/m^2^, *p* = .053). In the T1D group median FGF23 was not significantly correlated with BMI; however, in the control group we observed that median FGF23 was positively correlated with BMI (*r* = 0.166, *p* = .004). In the univariate analysis logFGF23 was not significantly correlated with BMI in the T1D group nor in the control group.

#### 3.2.3 Hypertension

In the T1D group 105 of the 302 subjects were classified as having hypertension versus 35 of the 302 subjects in the control group (35% vs 12%). In the T1D group 70 of the 105 subjects with hypertension used angiotensin‐converting enzyme (ACE) inhibitors. In the control group all 35 subjects with hypertension used ACE inhibitors. Median FGF23 was not significantly different between subjects with or without hypertension in both the T1D and control group.

Median hear trate and systolic blood pressure were slightly higher in patients with T1D than in controls (75/min vs 72/min, *p* < .001 and 125 vs 122 mm Hg, *p* = .003 respectively). Median diastolic blood pressure was not significantly different between patients with T1D and controls. All median values for heart rate and blood pressure were within the normal range. In the T1D group current smokers had a slightly higher heart rate than nonsmokers (78/min vs 74/min, *p* < .05). We found the same result in the control group (75/min vs 71/min, *p* < .05).

#### 3.2.4 Dyslipidemia

In the T1D group 227 of the 301 subjects were classified as having dyslipidemia versus 191 of the 302 subjects in the control group (75% vs 63%). Values of the serum lipid profile in the T1D group were available for 301 subjects. In the T1D group 31% of the subjects reported the use of statins versus <3% in the control group. Median TC, LDL‐c, and TG were all significantly lower and median HDL‐c was significantly higher in the T1D group than in the control group (see Table 1).

Median FGF23 was positively associated with TG (*r* = 0.146, *p* = .011) and TC (*r* = 0.114, *p* = .047) in the T1D group, these associations were not observed in the control group. When we performed the univariate analyses with log‐transformed data we could not reproduce these associations. Median FGF23 was not significantly correlated with LDL‐c or HDL‐c in both the T1D group and the control group.

In the T1D group median FGF23 was significantly higher in subjects with dyslipidemia than in subjects without dyslipidemia (78.6 vs 67.9 U/mL, *p* = .032). This difference was not present in the control group.

#### 3.2.5 Kidney function

In the T1D group 21 of the 302 subjects had an impaired kidney function (eGFR <60 mL/min/1.73m^2^) versus 15 of the 302 subjects in the control group (7% vs 5%). Median eGFR was not significantly different between the T1D group and the control group. Median FGF23 was not significantly different between subjects with or without an eGFR <60 mL/min/1.73m^2^ in both the T1D and control group. In our population there was no correlation between FGF23 and eGFR, with eGFR >60 mL/min/1.73 m^2^ for nearly all subjects.

#### 3.2.6 Urine albumin/creatinine ratios

There were substantial missing data for the urine samples in the T1D group; results were available for only 130 subjects. In the T1D group 14 of the 130 subjects had an elevated UACR (>3.0 mg/mmol) versus <10 of the 302 subjects in the control group (12% vs <3%). Median FGF23 was not significantly different between subjects with or without an elevated UACR in both the T1D and control group.

#### 3.2.7 Multivariable regression analyses

Multivariate analysis showed significant associations of sex, serum phosphate, alkaline phosphatase, and PTH with logFGF23 in the T1D group. In the T1D group the value of logFGF23 can be explained for 13.4% by sex, HbA1c, serum phosphate, serum calcium, serum alkaline phosphatase, and PTH (Table 3).

**TABLE 3:** Multivariate regression analysis of logFGF23 and other parameters in T1D group.

Variables	ß	SE	*p* value
Sex	0.069	0.024	**.005**
HbA1c	0.018	0.010	.080
Phosphate	0.139	0.052	**.008**
Calcium	0.244	0.134	.069
Alkaline phosphatase	0.001	0.000	**.028**
PTH	0.017	0.008	**.028**

*Note*: ß is beta coefficient. Bolded values are *p* < .05.

Abbreviations: FGF23, fibroblast growth factor 23; HbA1c, glycosylated hemoglobin; PTH, parathyroid hormone; T1D, type 1 diabetes.

## 4 DISCUSSION

This is the first study that evaluated FGF23 in patients with T1D mellitus and compared the data with age‐ and sex‐matched controls. It is important to get more knowledge about the pathogenesis of cardiovascular complications of T1D because it is the main cause of death.

In the present study serum calcium, phosphate, CPP, and alkaline phosphatase levels were higher in patients with T1D than in controls and were positively correlated to FGF23. A large study in the general population showed that higher serum phosphate levels were associated with coronary artery calcifications.^31^ The association was present in the absence of CKD, hyperphosphatemia, and prevalent CVD. Several studies have demonstrated that serum calcium, phosphate, and calcium‐phosphate product levels are associated with an increased risk of CVD. Shin et al^32^ found that, in a large study with subjects undergoing coronary disease assessment for a variety of clinical indications, serum calcium, phosphate, and calcium‐phosphate product levels are independent risk factors of coronary atherosclerosis, even in subjects with near‐normal kidney function. Foley et al^33^ found that, in a community‐based epidemiological study of CVD in the United States, serum calcium, phosphate, and calcium‐phosphate product were risk factors for stroke and death after more than 12 years of follow‐up. Ramírez‐Morros et al^34^ found that, in adults with type 2 diabetes, subjects with subclinical carotid atherosclerosis (SCA) had higher serum phosphate levels and a higher calcium‐phosphate product versus subjects without SCA. They did not find a significant difference in serum calcium between the two groups.

Females with T1D had higher FGF23 levels than males with T1D. Our result is in line with previous studies.^15^, ^26^, ^35^ Ix et al^35^ found that, in community‐living individuals with prevalent CVD, older women (mean age 67 ± 11 years) who were not using estrogen had higher FGF23 levels compared to either women using estrogen or men in a similar age group. They suggest that FGF23 levels may be influenced by postmenopausal changes. A possible mechanism might be the decreasing estrogen levels in menopause, but this is not yet fully understood and data are conflicting. Koek et al^36^ have investigated the calcium‐phosphate metabolism in men and women. They found that women ≥45 years of age had higher serum calcium and phosphate levels compared to men with the same age. They also suggest that postmenopausal changes might lead to this effect. These findings may be of relevance for increased incidence of CVD in women after menopause. Unfortunately, we did not have information on current estrogen levels in females nor did we have information about the possibility of current menopause or postmenopausal state.

Serum calcium, phosphate, CPP, and alkaline phosphatase were positively correlated to logFGF23. Correlations between FGF23 and serum calcium and phosphate have been described in previous research. Yasin et al^37^ found that, in their study with 81 children and young adults with different stages of CKD, FGF23 was positively correlated with phosphate and calcium‐phosphate product in the group of patients older than 12 years of age. They did not find this correlation in the younger children. They also found serum calcium levels in the normal range for all CKD stages. Serum phosphate was elevated only in CKD stage IV. Kojima et al^38^ found that, in their group of 89 diabetic and nondiabetic patients on hemodialysis, log‐transformed FGF23 was positively correlated with serum calcium, phosphate, and calcium‐phosphate product. In the baseline characteristics they described normal levels of serum calcium and phosphate.

We found that current smokers had higher FGF23 levels compared to nonsmokers in the T1D group, and mean serum phosphate was not significantly higher. This difference was not present in the control group. Vervloet et al^39^ have described a positive association between FGF23 and smoking in a group of 604 patients with moderate to severe kidney disease. Among them were patients with and without diabetes; unfortunately, the type of diabetes was not provided. However, the association was strong and persisted after correcting for other well‐known cardiovascular and renal risk factors. They also provided several possible explanations for this association and concluded that it would be most likely that smoking reduces FGF23 sensitivity. In the study of Gutiérrez et al,^40^ among a group of community‐living men with largely preserved kidney function, smokers had higher FGF23 levels than nonsmokers. In line with these results is the study of Parker et al^41^ In their study of 833 participants of the Heart and Soul Study smoking was associated with higher plasma FGF23 concentrations. The higher FGF23 levels may contribute to increased risk of CVD in smokers with T1D. For a few years, anti‐FGF23 (burosumab) has been available as a therapeutic drug for X‐linked hypophosphatemia (XLH). In a patient with XLH and left ventricular dysfunction, the left ventricular function normalized during burosumab treatment. There are no data of treatment of patients with other diseases with heart function abnormalities related to high FGF23 levels.^42^

Previous studies have demonstrated that FGF23 levels are higher in obese individuals versus normal weight individuals and that BMI is positively correlated with FGF23 even in different study populations.^20^, ^28^, ^40^, ^43^, ^44^, ^45^ In our study we found these results only in our control group. Why we did not find these results in the T1D group may be due to the small amount of subjects with obesity (*n* < 50).

The influence of lipids on FGF23 in our study population is difficult to interpret because of the wide use of statins in the T1D group versus only one subject in the control group. That makes it difficult to compare the two groups. The use of statins may also explain why mean LDL‐c and TG levels were lower in the T1D group than in the controls.

Previous studies have demonstrated that FGF23 increases when eGFR decreases. In our study population there was no correlation between FGF23 and eGFR. This is probably due to the fact that eGFR was >60 mL/min/1.73 m^2^ for nearly all subjects.

### 4.1 Strengths and limitations

We conducted a case–control study to investigate FGF23, the calcium‐phosphate metabolism, and several cardiovascular risk factors in a large number of patients with T1D and controls without T1D. To this date very few studies on FGF23 in T1D patients are available. A limitation of our study is the cross‐sectional design. Together with the lack of data on cardiovascular events and mortality, it is not possible to evaluate causative mechanisms.

## 5 CONCLUSION

Serum calcium, phosphate, CPP, and alkaline phosphatase levels were higher in patients with T1D than in controls and were positively correlated to logFGF23 in patients with T1D. FGF23 was higher in females with T1D than in males with T1D. FGF23 was higher in current smokers than in nonsmokers with T1D. These findings may be related to the increased risk of CVDs in patients with T1D.

## FUNDING INFORMATION

This research did not receive any specific grant from any funding agency in the public, commercial or not‐for‐profit sector.

## DISCLOSURE

Annemieke M. Boot received a research grant of Ultragenyx and Kyowa Kirin and consulting fees of Kyowa Kirin (paid to the hospital). Martine T. P. Besouw received a speaker fee from Kyowa Kirin (paid to the hospital). The other authors have no conflicts of interest to report.
